# Rhythm of the First Language: Dynamics of Extracellular Vesicle-Based Embryo–Maternal Communication in the Pre-Implantation Microenvironment

**DOI:** 10.3390/ijms24076811

**Published:** 2023-04-06

**Authors:** Kasun Godakumara, Paul R. Heath, Alireza Fazeli

**Affiliations:** 1 Institute of Veterinary Medicine and Animal Sciences, Estonian University of Life Sciences, 50411 Tartu, Estonia; 2 Sheffield Institute for Translational Neuroscience (SITRAN), University of Sheffield, 385a Glossop Rd., Broomhall, Sheffield S10 2HQ, UK; 3 Department of Pathophysiology, Institute of Biomedicine and Translational Medicine, Faculty of Medicine, University of Tartu, 14B Ravila, 50411 Tartu, Estonia; 4 Academic Unit of Reproductive and Developmental Medicine, Department of Oncology and Metabolism, The Medical School, University of Sheffield, Sheffield S10 2RX, UK

**Keywords:** pre-implantation, embryo, trophoblast, endometrium, extracellular vesicles, RNA-based signalling, nanopore

## Abstract

One of the most critical steps in mammalian reproduction is implantation. Embryos with an impaired capacity for embryo–maternal crosstalk are thought to have a reduced potential for implantation. One agent of embryo–maternal communication is extracellular vesicles (EV). EVs are lipid bilayer-bound biological nanoparticles implicated in intercellular communication between many of the known cell types. In the current study, we isolated EVs from trophoblast analogue JAr spheroids and supplemented the EVs with receptive endometrium analogue RL95-2 cells to simulate pre-implantation embryo–maternal dialogue. The transcriptome of the endometrial cells was examined at 30 min, 4 h and 48 h intervals using Oxford Nanopore^®^ technology. At the time points, 30 min, 4 h and 48 h, the endometrial cells showed a significantly altered transcriptome. It seems trophoblast EVs induce a swift and drastic effect on the endometrial transcriptome. The effect peaks at around 4 h of EV supplementation, indicating a generalized effect on cell physiology. Alterations are especially apparent in biological pathways critical to embryonic implantation, such as extracellular matrix–receptor interactions and cytokine–receptor interactions. These observations can be helpful in elucidating the dynamics of embryo–maternal communication in the pre-implantation period.

## 1. Introduction

One of the most critical steps in mammalian reproduction is implantation. During the process, the embryo first adheres to the luminal epithelium of the uterus and then invades the underlying stroma. Implantation is also considered the bottleneck step in human-assisted reproduction, where two out of three of the failed attempts are caused by a failure to implant [1]. Despite tremendous advances in the field of assisted reproduction technologies (ART), the success rate of embryo transfer remains less than 50%, mostly due to the embryo’s inability to implant [2]. Deciphering the causes for this unique situation is a subject of vigorous scientific inquiry, given that the morphological quality of the embryo does not correlate completely with the rate of implantation or successful birth, even in cases where the uterine environment is optimal for implantation [3].

The endometrium undergoes multiple physiological and morphological changes to be receptive to incoming implantation during the short period known as the window of implantation (WOI) [4]. These changes extend to a complete re-organisation of the uterine immune micro-environment. Conventional wisdom suggests that the endometrium and embryo are both passive or “quiet” and all the events leading up to and during the implantation and invasion of the endometrium by the embryo are regulated by external factors such as hormones and cytokines. This is, in fact, proven scientific theory and is supported by multitudinous reports. However, in recent decades, evidence has started to point to another component of the regulatory mechanism of embryo implantation, embryo–maternal communication [5].

This hypothesis posits that the embryo and the endometrium do not passively follow the directives of the endocrine system but actively participate in the process by communicating with each other using one or more signalling methods [6]. Although endometrial regulation by endocrine agents such as oestrogen and progesterone is well known, it does not represent the complete picture of endometrial receptivity regulation. Another aspect of the regulation of implantation is the action of cytokines, which function in an autocrine or paracrine manner during the process of implantation [7]. For instance, leukaemia inhibitory factor (LIF) is a mediator of oestrogen activity and a member of the interleukin-6 family [8]. LIF is also reported to stimulate and regulate stromal proliferation via the epidermal growth factor (EGF) signalling pathway [9]. In addition, heparin-binding epidermal growth factor-like growth factor (HB-EGF) is another crucial molecule in the implantation process [10]. Functioning in a juxtracrine manner, HB-EGF is a transmembrane protein that releases a soluble growth factor that reacts with the EGF family receptors on the trophoblast surface to regulate adhesion [11,12,13].

Another less well-studied facet of embryo–maternal communication is the communication that is hypothesized to utilize extracellular vesicles (EV) as the mode of signal transfer. EVs are defined as membrane-bound, nano-sized particles produced by living cells and released into the extracellular space [14,15]. All of the known cell types across the biological kingdoms are reported to produce EVs [16]. Many biological fluids, such as serum, cerebrospinal fluid and seminal plasma, are known to contain EVs [17]. One of the primary functions of EVs is to transport biologically significant cargo between cells. The transfer is known to occur between cells of the same individual, between individuals and even cross-kingdom. The cargo encompassed in EVs is extremely varied and heterogeneous, even among EVs extracted from purified cell cultures [14].

The involvement of EVs in embryo–maternal communication, especially in the crucial stage of embryo implantation, is not a well-studied field. However, multiple studies report on the involvement of EVs in other aspects of mammalian reproduction, from gamete production and maturation to foetal–maternal communication [18,19]. Our own studies have previously shown significant EV-mediated embryo–maternal communication and the significant effects that such communications induce in endometrial cells [20,21,22,23,24,25,26]. We have termed these communications “the first language” since they are the first communication between the progenitor and the progeny. We have shown evidence that EVs take part in the “first language” via trophoblast EV-induced transcriptomic alteration in the endometrium, increasing the receptivity of the endometrium. However, the exact mechanisms that EVs utilize to induce these physiological effects are still unclear. In the current study, we endeavour to investigate the transcriptomic effects induced by trophoblast EVs in the endometrial cells as a function of time using mRNA sequencing.

## 2. Results

The current study was carried out to investigate the effects induced by trophoblast analogue 3D spheroid-derived EVs on endometrial analogue cell transcriptomes as a function of time. Since we have demonstrated that trophoblast spheroid-derived EVs can alter the endometrial transcriptome with 24 h incubation in our previous communications, we have selected 30 min, 4 h and 48 h incubation times for this study. After each incubation, the endometrial RNA was collected and sequenced to determine the effects of trophoblast spheroid-derived EVs on the endometrial transcriptome.

### 2.1. Trophoblast-Derived EVs Altered Endometrial Transcriptome Significantly in Each Tested Time Point

**Figure 1:**
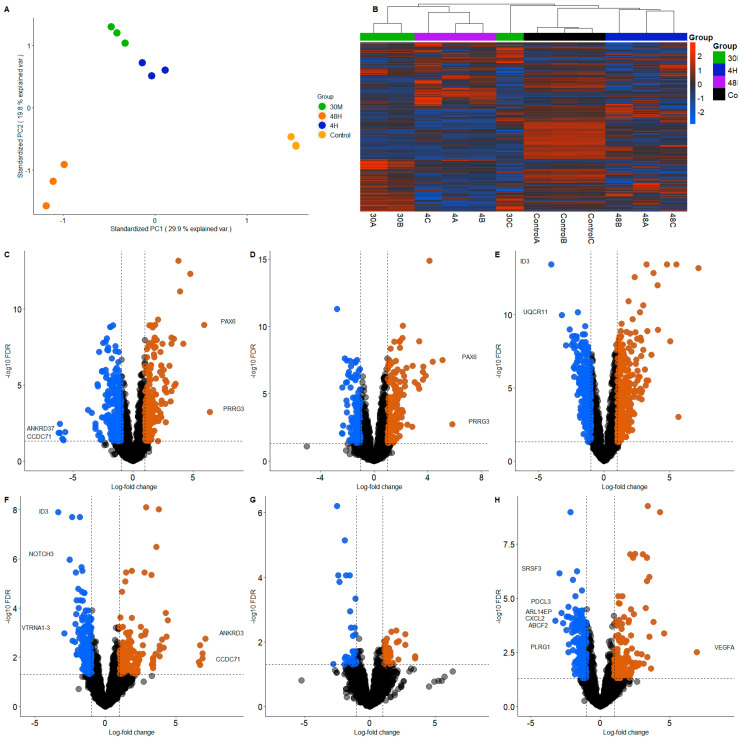
Receptive endometrial analogue RL95-2 cells were treated with trophoblast analogue Jar cell-derived EVs for 30 min, 4 h and 48 h to investigate the effect of trophoblastic EVs on endometrial transcriptome. (**A**) The PCA plot shows the variance between the groups (30 min; green, 4 h; blue, 48 h; orange and control; yellow). (**B**) Heatmap shows the differential expression of the genes (30 min; green, 4 h; blue, 48 h; purple and control; black). Volcano plots show the expression of individual genes in each comparison: 30 min treatment vs. control (**C**), 4 h treatment vs. control (**D**), 48 h treatment vs. control (**E**) 4 h vs. 30 min (**F**), 48 h vs. 30 min (**G**), 4 h vs. 48 h (**H**). Downregulation (FDR < 0.05, logFC < −1) is denoted in blue and upregulation (FDR < 0.05, logFC > 1) in orange.

The transcriptomes of endometrial cells treated with trophoblast-derived EVs were significantly altered at all the observed time points. The co-incubation time appeared to have a significant effect on the endometrial cellular transcriptome based on the principal component analysis (PCA) plot (Figure 1A) and the heatmap (Figure 1B), where the variability among the groups (30 min incubation, 4 h incubation and 48 h incubation, and the untreated control) are clearly visible. The untreated control (0 h) was used after observing the stable nature of the untreated RL 95-2 transcriptome as a function of time in cell culture (Supplementary File S1). No significant intra-group variation is apparent. The different altered transcriptomic profiles are presented in the volcano plots: 30 min treatment vs. control (Figure 1C), 4 h treatment vs. control (Figure 1D) and 48 h treatment vs. control (Figure 1E). In the 30 min vs. control comparison, there were 455 upregulated genes and 472 downregulated genes. In the 4 h vs. control comparison, there were 315 upregulated genes and 373 downregulated genes. In the 48 h vs. control comparison, there were 658 upregulated and 625 downregulated genes. In the 4 h vs. 30 min comparison, 272 genes were upregulated and 203 genes were downregulated (Figure 1F). In the 48 h vs. 30 min comparison, 53 genes were upregulated and 60 genes were downregulated (Figure 1G). In the 4 h vs. 48 h comparison, 314 genes were upregulated and 214 genes were downregulated (Figure 1H). A gene was deemed upregulated or downregulated when the log2 fold change (logFC) was more than +1 or less than −1, respectively. The only value of significance was the FDR (false discovery rate) < 0.05. Differentially expressed genes are presented in Supplementary File S2.

### 2.2. Different Sets of Genes Showed Altered Expression at Each Observed Time Point

**Figure 2:**
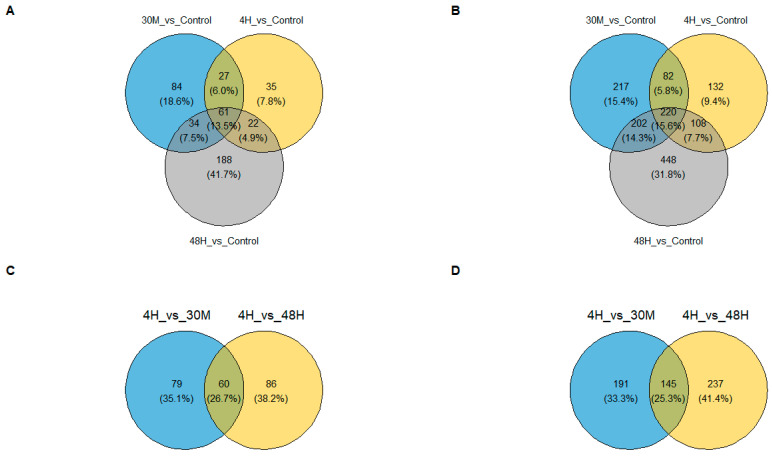
Receptive endometrial analogue RL95-2 cells were treated with trophoblast analogue Jar cell-derived EVs for 30 min, 4 h and 48 h to investigate the effect of trophoblastic EVs on endometrial transcriptome. (**A**,**C**) The Venn diagram depicts the relationship between the genes upregulated (FDR < 0.05, logFC > 1). (**B**,**D**) The Venn diagram depicts the relationship between the genes downregulated (FDR < 0.05, logFC < −1).

Observed transcriptomic alterations in endometrial cells treated with trophoblast spheroid-derived EVs were caused by different sets of genes at each observed time point. In upregulated genes (Figure 2A), only 61 genes (13.5%) were upregulated at all three time points. In downregulated genes (Figure 2B), only 220 genes, or 15.6% of the total genes, were downregulated in all three conditions. When considering 4 h vs. 30 min and 48 h vs. 30 min comparisons, there is a slightly higher number of jointly upregulated (60 genes; 26.7%) and downregulated (145 genes; 25.3%) genes. A gene was deemed upregulated or downregulated when the log2 fold change (logFC) was more than +1 or less than −1, respectively.

### 2.3. Pathways Crucial in Embryo Implantation Were Enriched in a Specific Pattern along the Three Observed Time Points

**Figure 3:**
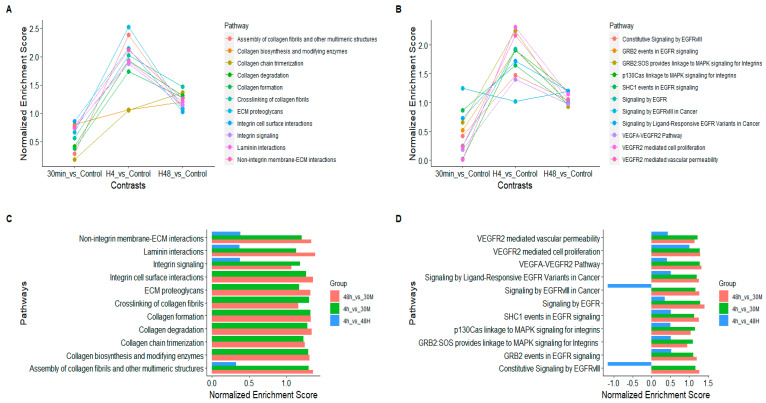
Receptive endometrial analogue RL95-2 cells were treated with trophoblast analogue Jar cell-derived EVs for 30 min, 4 h and 48 h to investigate the effect of trophoblastic EVs on endometrial transcriptome. Resulting transcriptomes were subjected to gene set enrichment analysis to identify enriched Reactome pathways. (**A**,**C**) The relative enrichment of pathways belonging to the general theme of extracellular matrix reorganization at each time point. (**B**,**D**) The relative enrichment of pathways belonging to the general theme of EGFR-based signalling at each time point.

Gene set enrichment analysis revealed that a group of pathways associated with the reorganization of the extracellular matrix (Figure 3A,C) and signalling with the epidermal growth factor receptor (Figure 3B,D) were significantly enriched in all three observed time points. The significance of enrichment was assumed when the adjusted *p*-value < 0.05. Interestingly, the normalized enrichment scores (NES), which denote the level of enrichment in each pathway in terms of the fold changes in the participating genes, followed a specific pattern in both groups of the considered pathways. The highest NES were observed at the 4 h time point, implying a greater amount of specific transcriptomic alterations. At the 48 h time point, the pathways showed a clear return to the base levels. All of the enriched pathways are presented in Supplementary File S3.

### 2.4. The Expressions of Key Genes in the Selected Pathways Were Significantly Altered Due to the Influence of EVs

**Figure 4:**
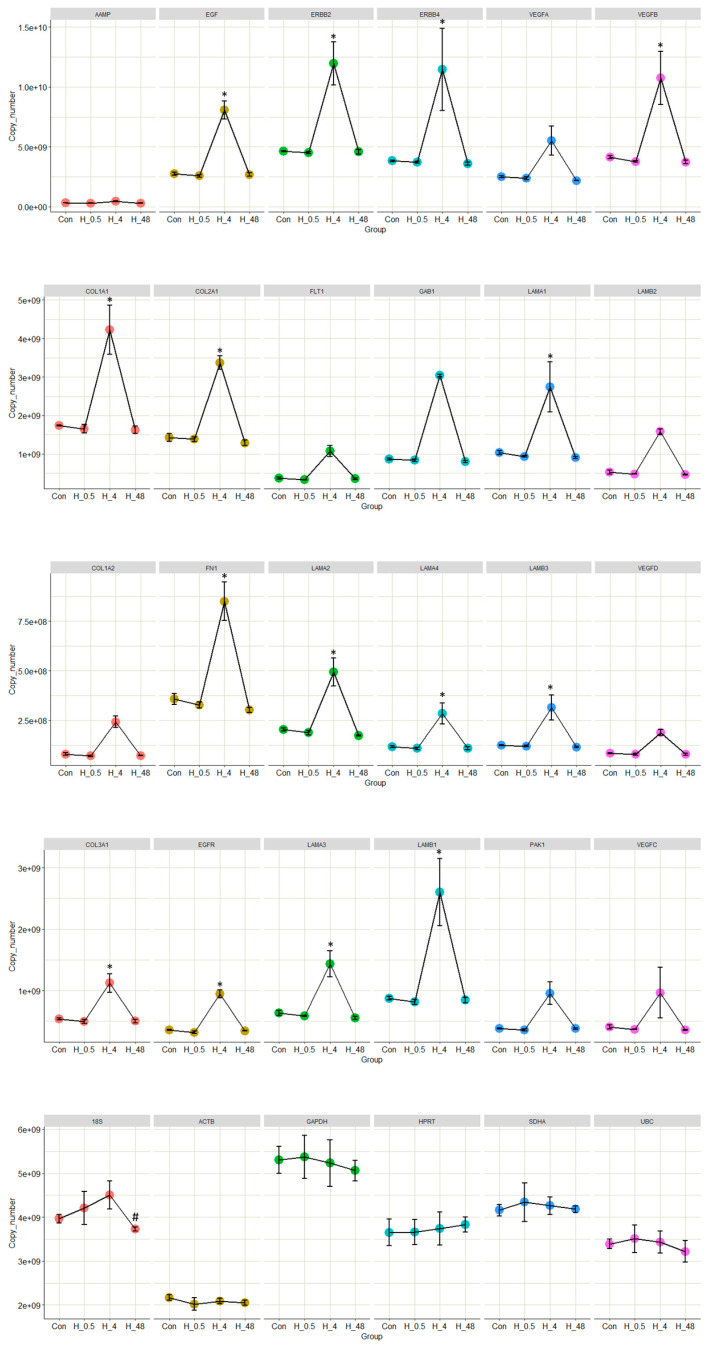
Receptive endometrial analogue RL95-2 cells were treated with trophoblast analogue Jar cell-derived EVs for 30 min, 4 h and 48 h to investigate the effect of trophoblastic EVs on endometrial transcriptome. RNA isolated from RL 95-2 cells was subjected to quantitative real-time PCR for key genes that are known to play critical roles in either extracellular matrix reorganization or signalling by EGFR. Expressions of five housekeeping genes were also measured to ascertain the stability of the RL 95-2 transcriptome during the period of incubation. Results are presented as absolute copy number vs. the group (30 min incubation, 0.5 H; 4 h incubation, H_4; 48 h incubation, H_48; Con, untreated control). *, *p* < 0.05 vs. untreated control. #, *p* < 0.05 vs. 4 h incubation.

The expressions of several genes that are reported to play key roles in either ECM reorganization or EGFR-based signalling were quantified using qPCR. In most cases, significant upregulations were observed in the endometrial cells incubated for 4 h with trophoblast-derived EVs compared to untreated controls. Interestingly, there were no significant upregulations or downregulations in the 30 min incubation group or the 48 h incubation group, either compared to untreated controls or each other (Figure 4). Five housekeeping genes were used to investigate the stability of the endometrial transcriptome during the period of incubation. There were no significant alterations to the expressions of the housekeeping genes. The measurement of significance was *p*-value < 0.05 from a single factor ANOVA.

## 3. Discussion

In our previous communications, using the same in vitro system as an analogue for the embryo–maternal microenvironment during the pre-implantation period, we observed drastic alterations to the endometrial transcriptome induced by the trophoblast-derived EVs. These observations implied that EVs play a major role in embryo–maternal communication. When considering the pathways enriched in endometrial cells treated by EVs, we were able to conclude that EV-mediated embryo–maternal communication increases the endometrial receptivity, priming the endometrium for the incoming embryo [22,23]. However, the mechanism of the communication was not apparent from one snapshot of the endometrial transcriptome taken at 24 h of EV–cellular co-incubation. The current study was carried out to create a more holistic image of the dynamics of EV-induced transcriptomic alterations in endometrial cells.

Significant alterations to the endometrial transcriptomes were observed in all three tested time points. Zero-hour untreated controls were used to compare the transcriptomic alterations at each time point after confirming that the RL95-2 transcriptome remains stable during 48 h of cell culture (Supplementary File S1). This observation is especially interesting since the first time point was a mere 30 min post-EV supplementation. The extremely short amount of time required to induce the transcriptomic alterations hints at a very quick mechanism of action in terms of signal transfer.

There are a few methods thought to be used by EVs in intercellular communications. The most tested hypotheses include EV-based communication via EV cargo transfer. EVs are known to carry a large variety of biologically active molecules such as proteins, lipids and RNA that are thought to be released into the cytoplasm of the target cell and bring about the physiological effect [27]. This premise calls for a method of EV uptake and cargo release. It is hypothesized that EVs either fuse directly to the plasma membrane of the target cell and release the cargo or the target cell actively uptakes the EVs, which are subsequently degraded to release the cargo [28,29,30].

Given the time taken for EV uptake, vesicle degradation and cargo release [30,31,32], plus the time that it takes to induce the transcriptomic alteration in the endometrial cells, it is unlikely that any mechanism that involves cargo release is responsible for the majority of the observed changes in the endometrial transcriptome at the 30 min time point. However, there is a higher possibility that EV-based communications that involve EV uptake and cargo release are responsible for at least a part of the endometrial transcriptome alterations observed at the 4 h and 48 h time points. The amount of commonly expressed transcripts (either upregulated or downregulated) at all time points is relatively low, implying different mechanisms are responsible for the changes in the transcriptome in each occurrence.

In our previous studies, we tried to correlate the embryonic miRNA carried in the EVs to the expression of their targets in the endometrium, working on the premise that EV miRNA is released to the cytosol of the receptor cell and downregulates their target mRNA. However, we were only able to correlate a minority of transcriptomic alterations (5–10%) to the actions of miRNA carried in EVs as cargo [22,23]. This observation and the short time taken to induce the transcriptomic effects, coupled with the reports that claim EVs are a very poor source of functional RNA and the very low percentage (1–2%) [30,33,34] of EVs whose cargo is released to the cytosol, leads us to deduce that cargo carried in EVs would be responsible for a minority of the transcriptomic effects observed in endometrial cells treated with trophoblast-derived EVs.

There are speculations about an intercellular signalling method involving EVs that does not involve EV uptake or cargo delivery. EVs are thought to bind to the plasma membrane using specific receptor–ligand complexes and initiate a signalling cascade that achieves the relevant physiological effect in the target cell [35,36,37,38,39,40]. Considering the rapidity of the trophoblast EVs’ effect on endometrial cells, it can be proposed that the initial transcriptomic alterations are achieved using a membrane-bound signalling system involving EVs. The signalling can also take place through the endosomal membrane once the EVs are taken up by the target cell via any of the endocytosis pathways.

Considering the pathways that are significantly enriched by the altered transcriptomes at each time point, gene set enrichment analysis revealed the over-representation of two groups of pathways: extracellular matrix (ECM) reorganization and pathways associated with epidermal growth factor receptor (EGFR)-related signalling. Both of these groups have been reported to be critically important in the implantation process. ECM remodelling is a critical change that the endometrial cells undergo in the process of decidualization. The receptors for the adhesion molecules are overexpressed on the apical surface and the pinopodes to facilitate trophoblast adhesion [41,42,43]. Major subunits of the ECM pathway, such as laminin interactions (R-HSA-3000157) [44], integrin cell surface interactions (R-HSA-216083) [45,46,47] and non-integrin membrane–ECM interactions (R-HSA-3000171) [48], are also significantly enriched and are all known to be implicated in the endometrial modifications in the WOI.

The epidermal growth factor receptor (EGFR) is a transmembrane protein belonging to the epidermal growth factor family, a subfamily of four closely related tyrosine kinases (EGFR (ErbB-1), HER2/neu (ErbB-2), HER3 (ErbB-3) and HER4 (ErbB-4)) [49]. They are reported to be critical for a successful early pregnancy, especially in regulating decidualization. EGFR is active during the window of implantation, and the impaired activation of such has been linked to many birth defects, such as intrauterine growth restriction and low birth weight [50,51,52,53]. Growth factors such as EGF and transforming growth factor alpha (TGFα) are implicated in mitogenic action, and the ablation of EGFR leads to severe subfertility in mice [49]. The cause of this subfertility is reported to be due to a failure in the maintenance and progression of the decidua [49,54].

Apart from being simply significantly enriched, the pattern exhibited by the level of enrichment, denoted by the normalized enrichment score (NES) in pathways associated with ECM- and EGFR-based signalling, is of special interest. Most pathways were enriched only mildly at the 30 min time point (NES between 0.3 and 0.9), implying a mild physiological effect. At the 4 h time point, a more drastic enrichment was observed, with enrichment scores of more than 1.5 in many cases. The cellular physiology seemed to return to its base physiology (denoted by 0 NES) at the 48 h time point. In our previous experiments, with 24 h co-incubation, we observed the NES to be around 1.3 to 1.6 for ECM-related pathways.

Another facet of the observed significant effect of trophoblast cell-derived EVs on the endometrial transcriptome can be perceived by contrasting the transcriptomes between the time points. The 4 h vs. 30 min and the 48 h vs. 30 min comparisons show that pathways are very similarly enriched on both occasions (Figure 3C,D). This can be interpreted as both the 4 h and 48 h transcriptomes being similarly altered compared to the transcriptome of the 30 min time point. Along with the fact that 4 h vs. 48 h shows very low amounts of pathway enrichment, these observations reaffirm the previous elucidation that there is a significant enrichment of critical pathways in embryo implantation as early as 30 min of trophoblast EV introduction, which increases by 4 h and remains in a slightly elevated status at the 48 h time point. Some pathways are even elevated in 48 h compared to 4 h, indicating lasting effects on endometrial transcriptome well beyond the half-life of EVs in cell culture conditions.

Quantitative real-time PCR results of 25 selected genes representing both pathway clusters showed a similar trend in expression profiles compared to the sequencing data. Most of the genes were very slightly differentially expressed from 0 h to 30 min, there were significant upregulations in most genes at the 4 hr time point and at 48 h the expression returned generally to the levels of 30 min and 0 hr. None of the genes showed any significant differential expression in the 48 h vs. 30 min comparison, indicating both the drastic nature of the transcriptomic changes seen at the 4 h time point and the fact that the endometrial transcriptome returned to a semblance of the base level after 48 hrs. All the selected genes were reported to regulate some critical function in embryo implantation, either as individual genes or as part of a pathway [49,55,56,57,58,59,60,61]. Interestingly, none of the genes that were upregulated or downregulated in all three time points nor the pathways they enrich were mentioned regarding human embryo implantation in previous publications, to the best of our knowledge (Supplementary File S4).

Taken as a whole, these observations suggest an underlying timeline of EV-induced transcriptomic changes, the mechanisms of which can be considered in the following manner. The minor pathway enrichments at the 30 min time point are the results of transcriptomic changes brought about by the swifter membrane-bound signalling accomplished by molecules on the EV surface binding to the receptors on the endometrial cellular plasma membrane. Then, at the 4 h time point, both the surface signalling and the effects of EV uptake and cargo release are seen as a drastic upregulation of implantation-critical pathways priming the endometrium for the incoming embryo. This heightened level of receptivity continues until the implantation allies with a constant source of embryonic EVs, such as an embryo that is endeavouring to implant. However, since we only introduced EVs once to the system, the transcriptomic changes began to plateau around 24 h post-EV treatment, and the endometrium returned to a slightly elevated level in 48 h. These observations are useful not only in elucidating the physiological effects of EVs but also in determining the half-life of EVs once introduced to the target cell culture.

The exact mechanisms/methods utilized by trophoblast-derived EVs to bring about the observed transcriptomic alterations can only be speculated based on the results of this study. Given the rapidity of the change in the endometrial transcriptome, the theory that posits that EVs contain membrane-bound signalling molecules that can initiate a cascade of events that eventually lead to the observed transcriptomic alterations would be the most educated guess at the present. However, there is very minimal evidence of any such occurrence in any system thought to be utilizing EV-based intercellular communications. Since both pathway clusters can be activated by a multitude of activators, it is not possible to accurately predict the correct molecule/s that may be responsible for the observed effects. Studies into the proteomes of trophoblast-derived EVs might be the next logical step in discovering the elusive agent of EV-mediated embryo–maternal communication.

The in vitro model used to mimic the pre-implantation microenvironment was made from cancer-derived cell lines. However, the model has been in use since the 1970s and is widely regarded as one of the best representations of the pre-implantation microenvironment. Given that using embryos and receptive endometrial tissue for experimentation is highly questionable ethically, the in vitro model was seen as an acceptable alternative.

The sequencing platform Oxford Nanopore^®^, although designed especially for ultra-long length reads, is an admirably user-friendly and low-cost method to investigate the differential expression of cellular mRNA. The obstacles introduced by the shallow depth of reads were mitigated with multiple wash/reload cycles, shorter read lengths and longer terms of sequencing. Even with extremely short read lengths (when considering the possibilities of Oxford Nanopore^®^, Littlemore, Oxford, UK), the coverage of the transcriptome was superior, in our hands, to Illumina sequencing. The Oxford Nanopore^®^ platform is much more cost-effective and less labour-intensive compared to sequence-by-synthesis methods.

## 4. Materials and Methods

### 4.1. Cell Culture and Trophoblast Spheroid Preparation

All the immortalized cell lines were purchased from the American Type Culture Collection in Teddington, United Kingdom. All the cell lines were cultured in a 37 °C and 5% CO_2_ environment. The endometrial analogue cell line was prepared using RL 95-2 cells (human adenosquamous carcinoma, ATCC CRL-1671). The culture medium for the RL95-2 cells consisted of Dulbecco’s Modified Eagle Medium (DMEM 12-604F, Lonza, Verviers, Belgium), 5 μg/mL human recombinant insulin (Gibco, Invitrogen, Denmark), 1% Penicillin-Streptomycin (P/S, Gibco™ 15140122, Bleiswijk, The Netherlands), 1% glutamine (Sigma, 59202C, Saint Louis, MO, USA) and 10% foetal bovine serum (FBS, Gibco™, 10500064). Cells were cultured at 37 °C and in 5% CO_2_.

The trophoblast analogue, human first-trimester choriocarcinoma cell line JAr (HTB-144 ™, Teddington, UK) cells, were cultured in RPMI 1640 (Gibco, Inchinnan, UK), 1% L-glutamine, 10% FBS, 1% penicillin/streptomycin. When the cells reached 80% confluency, they were collected using the trypsin-EDTA method, re-suspended in 5 mL of complete medium (1 × 10^6^ cells/mL concentration) and shaken overnight using a gyratory shaker (295 rpm, 37 °C, 5% CO_2_). The resulting spheroids were used as the analogues for the hatched trophoblast.

### 4.2. Depleting Extracellular Vesicles from FBS Using Ultrafiltration

In 2018, Kornilov et al. described a method of depleting EVs from FBS without compromising the material makeup of the reagent. We used the ultrafiltration method with modifications to deplete EVs from FBS that would later be used in the culture medium. FBS was centrifuged in a 100 kDa cut-off ultrafiltration unit (100 kDa, MERCK KGAA, Darmstadt, Germany) for 55 min at 3000× *g*. The filtrate was found to be up to 95% EV depleted [62]. Complete medium with EV-depleted FBS was prepared when trophoblast EVs were supplemented to endometrial cells.

### 4.3. Size Exclusion Chromatography for EV Enrichment

Size exclusion chromatography was used for trophoblast spheroid EV enrichment. Sepharose beads (cross-linked 4% agarose matrix of 90 µm beads) were used (Sepharose 4 fast flow™, GE HealthCare Bio-Sciences AB, Uppsala, Sweden) in a 10 cm gravity flow column. A 500 µL sample was added to the top of the column and fractions 7 to 10 of 500 µL fractions were collected in DPBS. Collected fractions were then further concentrated using 10 kDa ultrafiltration units.

### 4.4. RNA Extraction and Quality Control

RNA was extracted using the phenol–chloroform method with TRIzol reagent acting as the chaotropic agent (TRIzol^®^ reagent; Invitrogen, Inchinnan, UK). Concentrated EVs or adhered cells were used as the RNA source. In the case of EVs, the TRIzol reagent was added directly to the solution. In the case of adherent cells, the conditioned medium was removed, and then the TRIzol reagent was added directly to the cell layer. The samples were then left at room temperature for 10 min to completely dissociate the nucleoprotein complexes, and 300 µL of chloroform for 1 mL of TRIzol was added. The samples were then vigorously shaken for 15 s and centrifuged at 12,000× *g* for 15 min at 4 °C. When the phases were separated, the aqueous phase containing the RNA was removed, and the RNA was precipitated using 500 µL of isopropanol at room temperature for 20 min. To increase the efficiency of RNA extraction, 10 µg of glycogen (UltraPure™ Glycogen, 10814-010, Thermo Fisher Scientific, Bleiswijk, The Netherlands) was added to the mixture. Once the precipitation was complete, the samples were centrifuged at 18,000× *g* for 30 min at RT to pellet the RNA. The RNA pellets were washed three times in 70% ethanol and eluted in 20 µL of nuclease-free water. Quantity and the quality of RNA were measured using the Qubit HS RNA kit and the Agilent Pico 6000 kits (Agilent technologies, Santa Clara, CA, USA).

### 4.5. Library Preparation and RNAseq

Oxford Nanopore^®^ platform was used for RNA sequencing due to its utility and affordability. We used the MinION device with R9.4.1 (FLO-MIN106D) flow cells and the cDNA PCR Barcoding kit (SQK-PCB-109) with 100 ng of input RNA. The initial pore count of the flow cell was 897. Library was prepared to produce 500 bases per read (487 ± 56). Sequencing time was 72 h (with wash/refill cycles every 24 hrs). Twelve samples were run through the flow cell with indexing. Demultiplexing, quality control and adapter trimming were achieved through the Oxford Nanopore^®^ proprietary software.

The 72 h sequencing run produced 16.32 million reads (average reads per sample: 1.272 M ± 0.183 M). The proprietary file format FAST5 was basecalled to FASTQ files using the guppy algorithm. The reads were aligned to human genome assembly version GRCh38.108 using Homo_sapiens.GRCh38.108.gtf annotations and GraphMap algorithm. Read counts at the gene level were obtained by using featureCounts [63]. Genes with at least 10 counts for all the samples in at least one of the experimental groups were retained in the analysis for subsequent differential expression testing. Alignment data is presented in the Table 1. 

### 4.6. Differential Gene Expression Analysis and Network Analysis

Differential expression (DE) analysis was carried out in R version 3.6.1, using the edgeR package version 3.26.8 [64]. Tagwise dispersion estimates were obtained based on the trended dispersions, and statistical comparisons were performed using a generalized linear model followed by likelihood ratio tests, also accounting for the experiment batch. We considered the differential expression of genes with a false discovery rate (FDR) ≤ 0.05 to be statistically significant.

Principal components were calculated using prcomp function from the Stats package and visualized using the ggplot2 package [65]. The pheatmap package [66] was used for heatmap visualization with hierarchical clustering based on Euclidean distance.

Gene set enrichment analysis (GSEA) and pathway over-representation analysis were conducted using the ReactomePA package [67] and Reactome Pathway database annotations [67]. GSEA was used for full gene lists obtained from DE analysis that were ranked by −log10FDR × log2FC, where FDR denotes adjusted *p*-values and FC the fold change.

### 4.7. Quantitative Real-Time PCR with Absolute Quantitation

A mixture of random hexamer and oligo (dT) primers was used for cDNA synthesis from enriched RNA (SuperScript^®^ VILO™ cDNA synthesis kit, 11754050). cDNA products were amplified in EvaGreen assay system (Solis BioDyne, Tartu, Estonia) with the following program: 95 °C for 15 min, followed by 40 cycles at 95 °C for 20 s, 60 °C for 20 s, and 72 °C for 20 s. For melting curve analysis, the fluorescence signals were collected continuously from 65 °C to 95 °C at 0.05 °C per second.

For absolute quantitation, spike-in and normalizing of transcripts, 100 bp region from Isopenicillin N-CoA synthetase gene was used (Biomer.net company, Ulm/Donau, Germany, molecular weight: 32,239 g/mol, 100 pmol/µL). Synthetic RNA was serially diluted 20 times. For the first serial dilution, 1 µL of synthetic RNA was added to 39 µL RNase-free water to final concentration of 2.5 µM. Serial dilutions were prepared with a dilution factor of 4×. Serial dilutions were reverse-transcribed and amplified using real-time PCR, and the cycle threshold (Ct) values of dilutions were plotted against the copy number of the transcripts. Exponential calibration curve was fitted. In parallel, 1 µL of synthetic transcript was added to the sample during TRIzol RNA extraction, and then the Ct of synthetic RNA in this sample was assayed to calculate the RNA extraction efficiency and normalizing factor [22,68].

### 4.8. Experimental Plan

Trophoblast spheroids were prepared using the trophoblast analogue human choriocarcinoma JAr cell line. EVs were isolated from JAr cells cultured in EV-depleted media for 24 h. The EVs were co-incubated for 30 min (*n* = 3), 4 h (*n* = 3) and 48 h (*n* = 3) with a monolayer of RL95-2 cells. The ratio between the EVs and cells was 50:1. After the incubation, the RL95-2 cells were collected, and the cellular RNA was sequenced using Oxford Nanopore^®^ platform. Control samples were prepared using untreated RL 95-2 cells.

## 5. Conclusions

Taken as a whole, the observations on the dynamics of the transcriptomic changes in endometrial cells induced by trophoblast cells indicate that: (A) the transcriptome is altered very rapidly when in contact with trophoblast EVs, indicating a probable mechanism/signalling method that is implemented rather quicker than the more familiar “EV uptake → cargo delivery → physiological effect achieved by a cargo” pathway. (B) Pathways that are critical for embryo implantation are significantly enriched in all three observed time points, indicating a specific physiological purpose of EV-mediated embryo–maternal communication. (C) The pathways are enriched in a pattern that indicates the influence of EVs as a function of time, allowing the deduction of the half-life of EVs in a cell culture system. The exact EV-related mechanisms or substances that induce these transcriptomic changes are hard to speculate with the current knowledge. Further investigations into EV surface molecules may reveal the responsible underlying mechanism. Understanding the timeline of EV-mediated embryo–maternal communication will be beneficial in developing diagnostic and therapeutic applications for embryo-derived EVs.

## Figures and Tables

**Table 1:** Alignment details of the libraries used in the study.

Group	Total Alignments	Successfully Assigned Alignments	Percentage
*30A*	737,886	197,435	26.75
*30B*	724,495	248,661	34.32
*30C*	832,026	221,035	26.56
*4A*	803,439	214,825	26.73
*4B*	635,143	202,754	31.92
*4C*	790,801	242,175	30.62
*48A*	628,615	187,983	29.9
*48B*	747,331	242,578	32.45
*48C*	758,514	195,382	25.75
*ControlA*	809,428	224,117	27.68
*ControlB*	664,114	176,640	26.59
*ControlC*	641,522	184,343	28.73

## Data Availability

The data presented in this study are openly available in Sequence Read Archive (SRA). Reference number PRJNA948686.

## Supplementary Materials

The supporting information can be downloaded at: https://www.mdpi.com/article/10.3390/ijms24076811/s1.
